# Blood gene expression profiling in pediatric systemic lupus erythematosus and systemic juvenile idiopathic arthritis: from bench to bedside

**DOI:** 10.1186/1546-0096-12-16

**Published:** 2014-05-08

**Authors:** Mileka Gilbert, Marilynn Punaro

**Affiliations:** 1 Pediatric Rheumatology, University of Texas Southwestern Medical Center, Children’s Medical Center, Texas Scottish Rite Hospital for Children, Dallas, TX, USA

**Keywords:** Gene expression profiling, DNA microarray, Interferon, Interleukin-1, Systemic lupus erythematosus, Systemic juvenile idiopathic arthritis

## Abstract

Blood gene expression profiling has led to major advances in the field of rheumatology over the last few decades. Specifically, DNA microarray technology has been integral in increasing our knowledge of key players in the pathogenesis of some rare pediatric rheumatic diseases. Our group, using microarray analysis, identified the interferon (IFN) gene signature in pediatric systemic lupus erythematosus (SLE) and has published data that suggest high doses of intravenous corticosteroid treatment may have benefit over strictly oral regimens. Additionally, DNA microarray technology led to our discovery that the interleukin (IL)-1 gene signature is associated with systemic juvenile idiopathic arthritis (sJIA) and to the use of IL-1 blockade with anakinra in this disease. We also reported the biologic rationale for use of anakinra early in the disease course. Anakinra is now being used as first-line treatment in sJIA in multiple centers. Herein, we review how information obtained from blood gene expression profiling has changed our clinical practice.

## Review

In the last few decades, there have been major advances in understanding the biology and treatment of rheumatic diseases. Older genetic studies in humans and animal models identified candidate genes that might contribute to pathogenesis of disease; however, few studies translated to identify novel therapeutic targets in clinical trials. In the late 1980’s, experiments in human synovial tissue explants identified tumor necrosis factor (TNF) as an important cytokine in the pathogenesis of rheumatoid arthritis (RA) [1]. These studies led to the production of TNF blockers that are widely used today in the treatment of inflammatory arthritis and have changed the course of disease [2,3]. Subsequent work has led to the use of additional biologic agents in rheumatic diseases, including inhibitors of interleukin (IL)-1, IL-6, and targets of B and T cell production/function [4-7].

Despite these advances, most rheumatic diseases still lack specific diagnostic tests and optimal biomarkers. Much work remains to truly elucidate the biology and pathogenesis of these diseases. Blood gene expression profiling is a powerful and efficient tool in this regard. Older techniques, like Northern blot and reverse-transcription PCR, measure the expression of a few genes at a time. DNA microarray is one technique first used in the 1990’s for large-scale genomic comparisons [8,9]. DNA microarray refers to a technology in which DNA fragments are spotted onto a solid substrate, like a glass slide or nylon membrane, or DNA oligonucleotides are chemically synthesized on a substrate. This substrate can be used to analyze variation in the genome (genome-wide analysis) or for gene expression microarray. In gene expression microarray, messenger RNA is first isolated from a target sample, converted to complementary DNA (cDNA), fluorescently-labeled, and then hybridized to the DNA probe on the solid substrate. A laser scans the array to detect the fluorescent signal from hybridization. Bioinformatics tools calculate the amount of cDNA bound to the probe. Gene expression microarray technology can measure up to 50,000 messenger RNA transcripts at one time (reviewed in [10]). Technology has advanced to include analysis of gene regulation, genome-wide methylation signatures, and individual exons, which allows investigation of alternative splicing [11]. An alternative approach to hybridization-based gene-expression profiling techniques is using RNA sequencing techniques that enable millions of cDNA bases to be sequenced in a short amount of time [11]. Quantitative gene expression in different conditions is compared by microarray analysis to study specific diagnostic biomarkers, changes in gene expression in the course of disease, and changes in gene expression in response to treatment. Differential gene expression is then verified at the protein level by enzyme-linked immunosorbent assay (ELISA), protein microarrays, or other multiplexed assays [11]. This information can provide clues to the pathogenesis of disease.

Gene expression profiling to discover diagnostic and prognostic biomarker signatures was first developed in the field of cancer. Gene expression studies revealed differentially regulated gene transcripts in invasive melanoma, identifying a property of highly aggressive disease [12]. Additionally, DNA microarray identified subtypes of diffuse large B cell lymphoma based on gene expression indicative of different stages of B cell differentiation [13]. This technology has since been used to study a variety of disease processes [14,15].

We have been fortunate to have a very close and long-standing collaboration between our clinical team and laboratory colleagues. For many years, we have routinely collected standardized information and prospectively performed validated outcome measures on our pediatric Systemic Lupus Erythematosus (SLE) and Systemic Juvenile Idiopathic Arthritis (sJIA) patients seen in clinic, as well as performed serial gene expression profiling on their blood. This microarray analysis has been integral in xour study of rheumatic diseases and, in effect, has changed our clinical practice. Children offer unique advantages in the study of rheumatic disease because of lack of comorbid conditions and presentation of more aggressive disease. Herein, we review how blood gene expression profiling using DNA microarray analysis has helped our understanding of pediatric SLE and sJIA, and is now shaping our treatment of these diseases.

### Systemic lupus erythematosus

**Figure 1:**
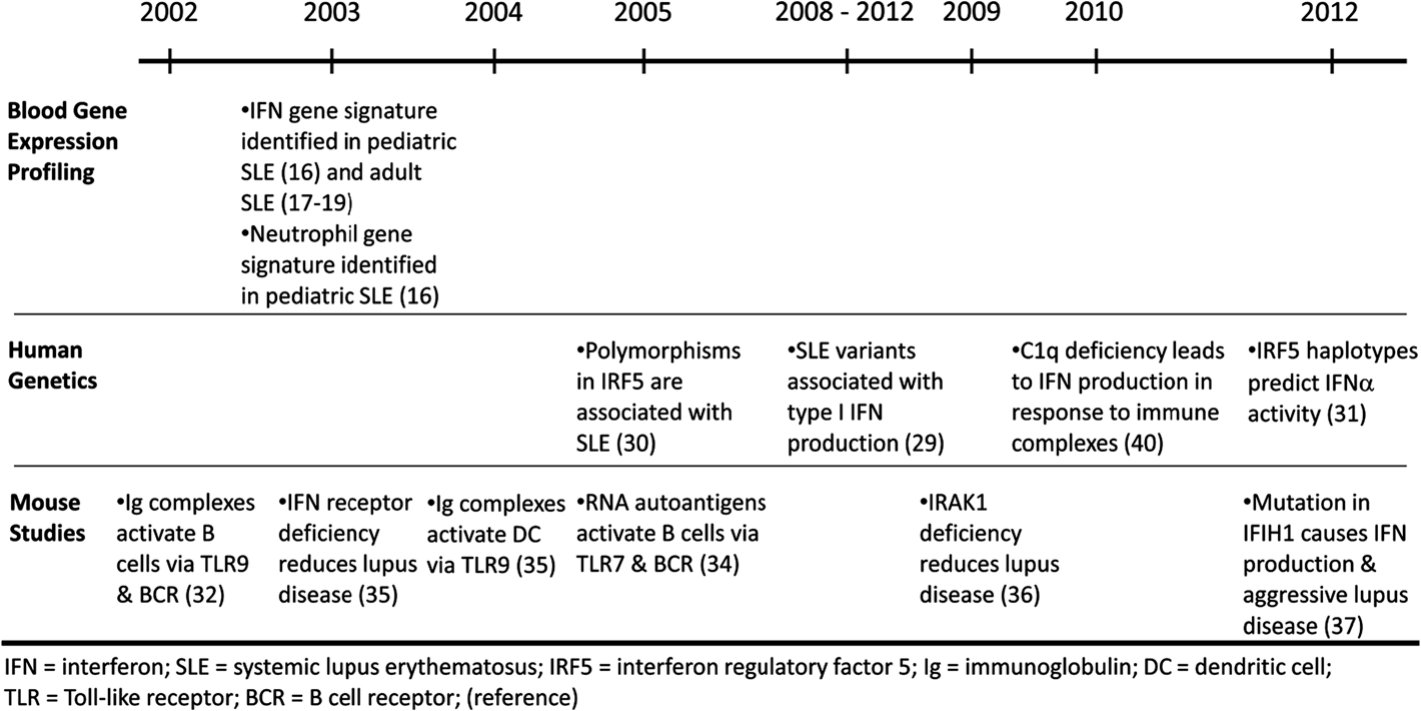
**Key Studies in the Role of IFN and Innate Immunity in SLE.** Timeline of publications implicating IFN and innate immunity in the pathogenesis of lupus.

SLE has been classically described as a prototypic autoimmune disease with a wide array of clinical manifestations and characterized by the production of autoantibodies to components of the cell nucleus. Consequently, components of the adaptive immune system were long the focus of research. More recently, the more sensitive test of gene expression profiling in peripheral blood mononuclear cells (PBMCs) has identified a significant role for components of the innate immune system in SLE. Using PBMCs rather than specific cell subsets allows for analysis of gene expression in a complex disease where there is interplay of multiple cell types. In 2003, our group was the first to report the presence of an interferon (IFN)-induced gene signature in the majority of our pediatric patients with active SLE [16]. In spite of heterogeneity of disease, there was a distinguishing homogeneous pattern of 14 upregulated genetic targets of type I IFN. Some of the 14 genes were related to known autoantigens in SLE and apoptosis. Other groups also reported the IFN-gene signature in adult patients with SLE in the same year [17-19] (See Figure 1 for key publications on the role of IFN in SLE).

The preeminence of the type I IFN signature in a disease previously considered to be a disorder of adaptive immunity, and in which genetic linkage and association studies had not, at that time, identified candidate lupus susceptibility genes within the IFN pathway, was surprising. However, a few previous observations suggested a role for dysregulated innate immunity and type I IFN in SLE. Some patients with lupus had been reported to have circulating IFNα [20]. Further, the sera of some SLE patients induced the differentiation of normal monocytes into dendritic cells (DCs), an effect which is mediated by IFNα [21]. DCs are critical for the maintenance of peripheral tolerance [22,23]; however, some patients with SLE have monocytes with properties of DCs that induced allogeneic T cell activation rather than tolerance [21]. Additionally, immune complexes of DNA and double-stranded DNA antibodies (normally found in SLE serum) induced plasmacytoid dendritic cells (pDC) to secrete IFNα [24]. Clinically, pDCs and IFN gene activation have been observed in inflamed tissues, skin lesions, and nephritic glomeruli from SLE patients [25-27]. IFNα treatment of patients with cancer and chronic viral infections induced autoantibody formation along with symptoms of SLE in a small percentage of patients [28].

Since 2003, several studies have supported the role of IFN in the pathogenesis of SLE. More recent studies have returned to the genetics approach in humans to identify genes associated with SLE that map into the type I IFN pathway (reviewed in [29]). An initial candidate gene study found an association of interferon regulatory factor 5 (IRF5) with SLE [30]. IRF5 is a transcription factor downstream of type I IFN and Toll-like receptor (TLR) signaling pathways. Certain IRF5 haplotypes are associated with serum IFNα activity in SLE [31]. Subsequent genome wide association studies have identified 47 independently confirmed SLE variants, 57% of which map in or near type I IFN or 7 genes with known key roles in type I IFN production or signaling, including *IRF5*, *IRF7*, *IRAK1*, *TNFAIP3*, *TNIP1*, *IFIH1*, and *TYK2* (reviewed in [29]). Many of these genes are also involved in TLR signaling pathways, an important link between innate and adaptive immunity.

Murine models of SLE have helped to support the role of IFN in lupus suggested by microarray and genetic studies. Several studies showed that immune complexes signal through TLR7/9 and initiate B cell and DC activation, leading to production of cytokines, including type I IFN, in lupus mice [32-34]. Deficiency in type I IFN receptor in NZBWF1 mice [35] and *IRAK1* (TLR signaling molecule) in a lupus-susceptible mouse [36] led to reduced lupus disease activity. Mutation in *IFIH1* gene (cytosolic sensor of dsRNA) triggered chronic type I IFN production and aggressive disease in lupus-susceptible, Fcγ receptor-deficient mice [37]. Microarray, genetic, and murine studies together provide an important link between TLR signaling and activation of type I IFN in SLE.

The description of monogenic lupus-related disorders has shed more light on the importance of the type I IFN pathway in the pathogenesis of SLE. Complement deficiencies, including C1q, were the first identified single-gene defects to cause lupus-like disease (reviewed in [38]). Patients with C1q deficiency develop lupus with high penetrance [39] and have high levels of IFNα in the serum and cerebrospinal fluid [40], presumably from defective clearance of apoptotic debris and lack of the inhibitory effect that C1q has on IFNα production by pDCs and monocytes [40]. Defects in DNase proteins also lead to the accumulation of extracellular DNA in apoptotic debris and are associated with SLE [41,42].

More recently, rare monogenic interferonopathies causing lupus-like disease have been described. Aicardi-Goutieres syndrome (AGS) is an early-onset progressive inflammatory encephalopathy with phenotypic similarity to both congenital infection and SLE [43]. AGS is caused by mutations in genes that encode nucleases. Defective function of these nucleases results in the intracellular accumulation of DNA, which can activate production of IFN in a TLR-independent manner. Blood gene expression profiling has revealed a type I IFN gene signature in patients with AGS [44,45]. The spectrum of disease associated with these mutations is not yet fully defined, but one study reports approximately 2% of SLE patients have mutations in the gene encoding the TREX1 exonuclease [46], mutations which can cause AGS. Mutation in *TREX1* is also associated with familial chilblain lupus, which is a nodular form of cutaneous lupus [47]. Another monogenic disorder involves mutation in tartrate-resistant acid phosphatase (*TRAP*), which causes spondyloenchondrodysplasia (SPENCD) syndrome, characterized by skeletal dysplasia, cerebral calcifications, and lupus-like autoimmunity [48]. TRAP dephosphorylates/inactivates osteopontin, which is involved in TLR9 signaling and type I IFN production in pDCs [49]. SPENCD patients have high levels of circulating IFNα and a prominent IFN gene signature [50]. These studies suggest IFN as a future target for therapy in the treatment of SLE and monogenic interferonopathies.

Clinically, it has been observed that 25% of SLE patients have endogenous anti-IFNα autoantibodies in the serum [51]. This subset of patients also had lower serum IFN levels associated with decreased IFN-pathway and disease activity, suggesting that neutralization of IFN activity may reduce disease activity in SLE. Therapies targeting IFN are currently being trialed in lupus patients (reviewed in [52]). Two IgG1 human monoclonal antibodies against IFNα have been developed (Sifalimumab and Rontalizumab). MEDI-546 is another monoclonal antibody that blocks the α subunit of the IFNα/β receptor. IFNα blockade significantly reduced the IFN-gene signature in patients, but no significant effects on disease activity have been observed so far [52]. An alternative approach is immunization with a synthetic compound made of IFNα2b molecules linked to a strong immunogenic carrier (IFNα kinoid), which induces the production of anti-IFNα autoantibodies. Immunization led to a polyclonal anti-IFNα response in patients, but no significant effect on disease activity [52]. Further studies on IFN blockade are necessary to determine its safety and efficacy in SLE.

In further support of the importance of the innate immune system in the pathogenesis of lupus, our 2003 study also identified a pattern of overexpressed granulopoiesis-related genes in SLE [16] (See Figure 1 for key publications on the role of innate immunity in SLE). This finding correlated with previous studies suggesting a role for neutrophils. Although numbers of circulating neutrophils in SLE are decreased, their presence in lesions of vasculitis and lupus nephritis is well described [53-55]. More recently, secretion of neutrophil-derived proteins in the urine has been shown to be a surrogate marker for active nephritis in pediatric SLE [56]. Treatment with granulocyte colony-stimulating factor (GCSF), which promotes granulopoiesis, may be associated with lupus flares [57]. In subsequent work to elucidate the role of neutrophils in lupus, we have demonstrated that anti-ribonucleoprotein antibodies found in SLE stimulate neutrophils to release extracellular traps composed of DNA and protein complexes [58]. These neutrophil extracellular traps (NETs) activate pDCs to produce high levels of IFNα in a DNA- and TLR9-dependent manner. Thus, neutrophils also contribute to the dysregulation of IFN in SLE.

Beyond highlighting the role of innate immunity in SLE, gene expression profiling also has therapeutic implications. When the IFNα signature was first recognized in the PBMCs of lupus patients [16], it was observed that the only therapy that extinguished the IFNα signature was high dose intravenous methylprednisolone (IVMP). It was also noted that the IFN signature returned in approximately one week after the IVMP was administered. Although the mechanism for this was not understood at that time, this observation changed our practice in treating pediatric patients with SLE. In association with other immunosuppression, we use frequent intermittent high-dose IVMP with low-dose daily oral steroids (rarely exceeding 10 mg/day) to induce remission in patients with severe systemic inflammation or major organ system involvement (CNS manifestations or lupus nephritis), and to treat severe flares of disease as well. Anecdotally, we have been impressed that this early intensive approach seems to limit the long term corticosteroid requirement and decrease steroid-related side effects in our patients.

Subsequently, the mechanism for this steroid resistance in SLE has been elucidated. Our group studied the effects of oral versus IV steroid regimens on the IFN signature in SLE using DNA microarray analysis [59]. Oral glucocorticoid therapy up to 20 mg daily dosing in lupus patients caused normalization of multiple transcriptional modules by microarray; however genes in the IFN pathway were not affected. In contrast, intermittent IVMP pulse therapy normalized the IFN signature. Typically, pDCs, the major source of IFNα production, are sensitive to steroid therapy [60,61]. However, ligation of TLR7 and 9 by self RNA and DNA in pDCs from lupus patients conferred increased survival and resistance of the pDC to glucocorticoid-mediated death. Higher steroid doses were able to overcome this resistance and extinguish the IFN signature by killing the pDC. The return of the IFN signature in one week coincided with the regeneration of the pDC population. The data suggest a biologic rationale for the use of intermittent high dose steroid therapy in the treatment of lupus.

Although steroids are used ubiquitously in SLE and cause significant morbidity, the use of corticosteroids in this disease is entirely empiric. Currently, there is no evidence base to support any specific doses, route of administration, or tapering schedules of corticosteroids in lupus. Since it is very unlikely that a randomized comparative trial will be performed to assess steroid regimens in SLE, the Childhood Arthritis and Rheumatology Research Alliance (CARRA) has developed consensus treatment plans for induction therapy of proliferative lupus nephritis with either mycophenolate mofetil or IV cyclophosphamide in combination with one of three options for standardized use of glucocorticoids [62]. These include a primarily oral, a mixed oral/IV, and a primarily IV regimen, the last of which is based on the science described above. The hope of the consensus treatment plans is to follow large numbers of pediatric SLE patients and eventually be able to compare effectiveness of the different therapeutic strategies in proliferative lupus nephritis.

### Systemic juvenile idiopathic arthritis

Systemic Juvenile Idiopathic Arthritis (sJIA), although historically classified as a subtype of juvenile arthritis, is easily distinguished from other subtypes of JIA by the presence of high grade fever and significant systemic inflammation, as well as the variable presence of evanescent rash, lymphadenopathy, serositis, enlargement of liver or spleen, and macrophage activation syndrome (MAS). The systemic features of sJIA may persist for months followed by the development of chronic arthritis. Up to 50% of children with sJIA have active arthritis as long as 10 years after diagnosis is made [63-65]. Also, sJIA is less responsive to standard therapies, such as methotrexate and TNF blockers, and more frequently requires the use of systemic steroids, in contrast to other JIA subtypes. Indeed, the long-term use of corticosteroids has been a major cause of morbidity in this condition. Thus, more specific biologic targets are necessary to treat disease and prevent morbidity caused by systemic steroids.

**Figure 2:**
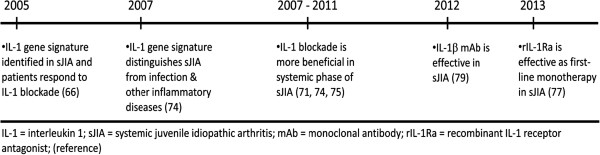
**Key Studies in the Dysregulation of IL-1 in Systemic JIA.** Timeline of publications describing the importance of IL-1 dysregulation in sJIA and treatment with IL-1 blockade.

In 2003, it was noted that the serum from 4 of our patients with sJIA, when cultured with PBMCs from healthy donors, upregulated the transcription of genes in the IL-1 cytokine/cytokine receptor family in addition to chemokines involved in chemotaxis of stem cells, neutrophils, monocytes/macrophages, lymphocytes, and DCs [66]. There was also increased secretion of IL-1β protein by healthy PBMCs cultured with sJIA serum. Thus, sJIA serum induced both transcription and translation of IL-1β in healthy PBMCs. Furthermore, activated PBMCs from sJIA patients in culture produced high levels of IL-1β, but not IL-6 or TNF, compared to healthy age-matched controls. These novel findings were published in 2005 and suggested that dysregulation of IL-1 production is central to the pathogenesis of disease, and that IL-1 blockade might be beneficial in our patients with sJIA [66] (See Figure 2 for key publications on the role of IL-1 in sJIA).

At that time anakinra, a recombinant human IL-1 soluble receptor antagonist (rIL-1Ra) with a short half-life of four to six hours was the only IL-1 blocker commercially available. Anakinra had initially been studied in adult patients with sepsis [67,68]. Large clinical trials were done using anakinra in doses up to 2 mg/kg/hr to study therapeutic efficacy and safety in septic shock. Anakinra did not improve survival but was found to be safe in this very sick population as it did not increase mortality or cause any additional adverse events compared to placebo. IL-1 blockade with anakinra had also been studied in large clinical trials of adults with rheumatoid arthritis (RA) [69] with a good safety profile. Thus, we decided to try anakinra in nine of our patients with sJIA who had active disease resistant to conventional aggressive treatment [66]. Seven of nine patients had systemic symptoms, and eight had active, uninterrupted arthritis with disease duration of 23–144 months. They were all being treated with oral prednisone, in addition to an IVMP regimen and/or methotrexate in the majority of patients. Some had previously failed treatment with TNF blockers, IVIG, and cyclosporin. Treatment with anakinra 2 mg/kg, up to 100 mg, in a daily subcutaneous injection resulted in a significant response in seven and a partial response in two of the nine patients. This dramatic clinical response to anakinra confirmed that IL-1 is an important mediator of sJIA. Since this study, similar responses have been seen in sJIA at other centers [70-72], and a good safety profile was demonstrated in a small trial of pediatric patients with polyarticular JIA [73].

A subsequent study first identified a sJIA-specific gene signature that uniquely differentiated sJIA from infection and other inflammatory diseases [74]. We compared gene expression in PBMCs from 44 sJIA patients in various stages of disease to children with acute infection, SLE, pyogenic arthritis pustulosis acne (PAPA) syndrome, as well as healthy controls. 88 sJIA-specific genes were identified, 12 of which accurately classified an independent set of sJIA patients with systemically active disease. An important aspect of this data was the demonstration that the sJIA signature was most evident during the systemic phase of the disease, which suggested that anakinra would be most useful during this phase of the disease.

As a result of this finding, our clinical practice in the treatment of sJIA changed. We now routinely use anakinra as first-line therapy, essentially as a steroid-sparing agent, either markedly reducing or eliminating our use of corticosteroids. In a recent retrospective chart review of our sJIA patients treated with anakinra for the past 10 years, 38/51 (75%) achieved inactive disease with 31/51 (60%) sustaining this state long enough to meet Wallace criteria for clinical remission on medication. Twenty-two of these patients were treated without any steroids (manuscript in preparation).

Gene expression in sJIA has been remarkably stable across different patient cohorts. Microarray analysis of PBMCs of sJIA patients in the ANAJIS trial showed significant upregulation of innate immunity and underexpression of adaptive immunity gene modules before treatment, similar to our Dallas sJIA cohort. The ANAJIS trial was a small multicenter, randomized, double blind, placebo-controlled trial done to study efficacy of Anakinra in treatment-resistant sJIA patients with active disease [75]. The 24 patients in this study were steroid-dependent with a minimum of six month disease duration. Active disease in the majority of patients at enrollment was characterized by polyarthritis and not fever, indicating that many patients were in the arthritic and not systemic phase of disease. Results showed anakinra treatment to be more effective in the short term. Additionally, several gene expression modules normalized at 1 and 6 months in those who responded to treatment with anakinra. These results corresponded with our prior work [74] and another study [71] that suggested anakinra may be more useful in the systemic phase of disease. Another observation of interest in the ANAJIS trial was that anakinra treatment also induced an IFNα gene signature.

Anakinra as a first-line therapy has also been embraced by other centers [76,77]. A recent prospective cohort study of 20 patients with steroid-naïve, new-onset sJIA showed that rIL-1Ra is effective in the majority of patients as monotherapy [77]. 75% of patients had an ACR Pediatric 90 response or inactive disease after three months and 65% after one year on rIL-1Ra alone. Approximately one-third of patients required additional medication to maintain clinical response. Treatment with rIL-1Ra could be stopped in the majority of responders within one year during a 32-month follow up period. Due to heterogeneity of disease, larger clinical trials are needed to study safety, appropriate dosing of anakinra, and predictors of response to anakinra in sJIA.

CARRA has developed consensus treatment plans and standardized assessment schedules for sJIA in clinical practice across North America [78]. Four standardized treatment plans were developed, one of which uses anakinra as first-line therapy with optional glucocorticoid treatment. The consensus treatment plans will allow the collection of data on large numbers sJIA patients treated with anakinra and other medications to evaluate comparative effectiveness in an observational setting. IL-1 blockade with IL-1β monoclonal antibody in two randomized-controlled trials has also been found to be effective in patients with active systemic features of sJIA concomitantly treated with glucocorticoids [79].

### Other inflammatory diseases

Pediatric rheumatologists care for patients with uncommon inflammatory diseases. There are cases where systemic inflammation is present without a clear etiology or definitive diagnosis. We have used DNA microarray analysis to help guide therapy in pediatric patients with inflammatory diseases of unclear etiology. We have followed an 8-year-old female patient with a history of recurrent strokes since 1 year of age, daily fevers up to 103°F, and livedo reticularis rash. Evaluation for infection, malignancy, and hypercoagulability was negative. She has had evidence of systemic inflammation, but no autoantibodies. It was suspected that she had a focal cerebral vasculitis/vasculopathy on imaging studies. The patient’s blood gene expression profile was analyzed by DNA microarray and revealed an IFN gene signature, similar to that found in SLE, but without overexpression of plasma cell genes. Thus, she was treated as a lupus-like condition with major organ involvement, with intermittent IVMP and IV cyclophosphamide treatment regimen transitioning to mycophenolate mofetil. Although her rash improved, she continued to have daily fevers. Subsequently, an IL-1 gene signature was identified in her blood, suggesting that she might have an autoinflammatory condition. IL-1 blockade was started with anakinra with initial improvement in fevers and no recurrence of stroke. The gene associated with her condition was recently identified as CECR1 (cat eye syndrome chromosome region, candidate 1) by whole-exome sequencing [80]. CECR1 gene encodes adenosine deaminase 2 (ADA2) protein. In a cohort of patients followed at the National Institutes of Health, loss-of-function mutations in CERC1 were associated with early-onset recurrent strokes, systemic vasculopathy and vasculitis. Knockdown of ADA2 homologue in zebrafish caused intracranial hemorrhage and neutropenia [80]. The function of this protein is currently under investigation. Our patient is currently managed in collaboration with the NIH with etanercept, anakinra, mycophenolate mofetil, IVIG, and oral prednisone to control symptoms.

## Conclusions

Although gene expression profiling has advanced our understanding of the basic biology of several rheumatic conditions and has led to more rational treatment strategies, challenges still exist in the field of pediatric rheumatology in the diagnosis and treatment of rare and chronic autoimmune and inflammatory diseases. Many diseases are diagnosed based on classification criteria that include nonspecific clinical and laboratory findings. Lack of specific diagnostic biomarkers often delays diagnosis and treatment, sometimes leading to severe complications. Disease course is then characterized by symptom flare and remission. We currently do not have objective measures of global disease activity or the ability to predict flares based on biology.

With the advent of DNA microarray technology, we are closer than ever before in identifying biomarkers for SLE and sJIA. Blood gene expression profiling has identified IFN and neutrophil-related genes signatures in SLE and IL-1 gene signature in sJIA. Another group has identified IFN-regulated chemokines as potential biomarkers of SLE disease activity [10,81]. Current research in our group is directed at finding biomarkers for the diagnosis of SLE and sJIA, monitoring the course of disease on treatment, and for the early prediction of disease activity flares. It is our hope that this work will ultimately lead to specific biomarkers that can be routinely used in clinical practice to diagnose disease, predict flares of disease activity, and to determine appropriate treatment.

## Abbreviations

SLE: Systemic lupus erythematosus; sJIA: Systemic juvenile idiopathic arthritis; IFN: Interferon; IL: Interleukin; TNF: Tumor necrosis factor; RA: Rheumatoid arthritis; ELISA: Enzyme-linked immunosorbent assay; PBMC: Peripheral blood mononuclear cell; pDC: Plasmacytoid dendritic cell; TLR: Toll-like receptor; IRF: Interferon regulatory factor; AGS: Aicardi-Goutieres syndrome; SPENCD: Spondyloenchondrodysplasia; GCSF: Granulocyte colony-stimulating factor; NETs: Neutrophil extracellular traps; rIL-1Ra: Recombinant IL-1 receptor antagonist; IVMP: Intravenous methylprednisolone; MAS: Macrophage activation syndrome; PAPA: Pyogenic arthritis pustulosis acne syndrome.

## Competing interests

The authors declare that we have no competing interests.

## Authors’ contributions

All authors prepared, read, and approved the final manuscript.

## Authors’ information

MG is a pediatric rheumatology fellow at the University of Texas Southwestern Medical Center. MP is the division chief of pediatric rheumatology at the University of Texas Southwestern Medical Center.
